# The Therapeutic Effects of Treadmill Exercise on Osteoarthritis in Rats by Inhibiting the HDAC3/NF-KappaB Pathway *in vivo* and *in vitro*

**DOI:** 10.3389/fphys.2019.01060

**Published:** 2019-08-20

**Authors:** He Zhang, Lu Ji, Yue Yang, Yingliang Wei, Xiaoning Zhang, Yi Gang, Jinghan Lu, Lunhao Bai

**Affiliations:** ^1^Department of Orthopedic Surgery, Shengjing Hospital, China Medical University, Shenyang, China; ^2^Department of Gynecology and Obstetrics, Shengjing Hospital, China Medical University, Shenyang, China; ^3^Department of Anesthesiology Department, Shengjing Hospital, China Medical University, Shenyang, China; ^4^Department of Orthopedic Surgery, Panjin Central Hospital, Panjin, China

**Keywords:** osteoarthritis (knee), HDAC3, inflammation, treadmill exercise, NF-kappaB, RGFP966

## Abstract

Osteoarthritis (OA) is a disease characterized by non-bacterial inflammation. Histone deacetylase 3 (HDAC3) is a crucial positive regulator in the inflammation that leads to the development of non-OA inflammatory disease. However, the precise involvement of HDAC3 in OA is still unknown, and the underlying mechanism of exercise therapy in OA requires more research. We investigated the involvement of HDAC3 in exercise therapy-treated OA. Expression levels of HDAC3, a disintegrin and metalloproteinase with thrombospondin motifs-5 (ADAMTS-5), matrix metalloproteinase-13 (MMP-13), HDAC3 and nuclear factor-kappaB (NF-kappaB) were measured by western blotting, reverse transcription polymerase chain reaction (RT-PCR) and immunohistochemistry. Cartilage damage and OA evaluation were measured by hematoxylin and eosin staining and Toluidine blue O staining according to the Mankin score and OARSI score, respectively. We found that moderate-intensity treadmill exercise could relieve OA. Meanwhile, the expression of HDAC3, MMP-13, ADAMTS-5 and NF-kappaB decreased, and collagen II increased in the OA + moderate-intensity treadmill exercise group (OAM) compared with the OA group (OAG) or OA + high- or low-intensity treadmill exercise groups (OAH or OAL). Furthermore, we found the selective HDAC3 inhibitor RGFP966 could also alleviate inflammation in OA rat model through inhibition of nuclear translocation of NF-kappaB. To further explore the relationship between HDAC3 and NF-kappaB, we investigated the change of NF-kappaB relocation in IL-1β-treated chondrocytes under the stimulation of RGFP966. We found that RGFP966 could inhibit the expression of inflammatory markers of OA via regulation of HDAC3/NF-kappaB pathway. These investigations revealed that RGFP966 is therefore a promising new drug for treating OA.

## Introduction

Osteoarthritis (OA) is a highly prevalent degenerative disease worldwide. In 2015, almost 54.4 million adults were diagnosed with OA in the United States (Cheng et al., 2010); this number is increasing rapidly. OA is a multi-factor disease, and obesity, age, gender, and abnormal loading of joints are all able to stimulate production of inflammatory mediators secreted by chondrocyte and synovium including IL-1β, ADAMTS-5 and MMP-13, which contribute to OA characterized by cartilage erosion, subchondral bone formation, and joint swelling (Ashkavand et al., 2013; Berenbaum, 2013; Mobasheri and Batt, 2016). Recently, exercise therapy has received considerable attention for treating OA, as an alternative to surgery (McAlindon et al., 2014). Gentle exercise can prevent subchondral cyst formation and osteoclast activity in OA development (Iijima et al., 2015); moderate-intensity exercise can also promote glycosaminoglycan (GAG) content in the cartilage and prevent cartilage damage including extracellular matrix loss, chondrocytes apoptosis, inflammation development, and osteophyte formation (Cifuentes et al., 2010; Ni et al., 2013; Boudenot et al., 2014). Conversely, improper exercise can aggravate OA (Franciozi et al., 2013). However, the mechanism of different intensities of exercise involved in the variable effect on OA is still unknown.

Histone deacetylases are a family of proteins that regulate acetylation of proteins, and can be divided into four groups: class I HDACs, class II HDACs, Sirtuins and HDAC11 (De Ruijter et al., 2003). HDACs largely contribute to the pathological process of OA; levels of HDAC1, 2 are elevated in the OA cartilage (Carpio and Westendorf, 2016), and HDAC4 can promote MMP-13 expression in OA (Song et al., 2015). As a member of class I HDACs, HDAC3 plays an important role in the development of normal cartilage and chondrocyte (Carpio et al., 2016), and also contributes to inflammatory diseases such as carditis (Zhu et al., 2010), spinal injury (Chen S. et al., 2018), and macrophage inflammation (Chen et al., 2012). Regarding arthritis, most studies have focused on the involvement of HDAC3 in leptin-induced arthritis and synovitis in rheumatic arthritis, caused by metabolic dysfunction and autoimmunity dysfunction, respectively (Chang et al., 2015; Angiolilli et al., 2017; Su et al., 2018). Only one article reported elevated HDAC3 was found in the cartilage of OA patients (Meng et al., 2018). Therefore, the role of HDAC3 in OA pathogenesis and exercise therapy requires further investigation.

Nuclear factor-kappaB (NF-kappaB) is a transcription factor that plays an important role in inflammation, especially in OA. NF-kappaB consists of five subunits, and the most common type is the p65/p50 heterodimer. Upon stimulation by pro-inflammatory factors, NF-kappaB becomes localized to nucleus and phosphorylated, promoting the expression of MMPs and ADAMTS, which result in degradation of cartilage matrix and OA progression. HDAC3 could regulate NF-kappaB activity. Alteration of acetylation of NF-kappaB by HDAC3 may modulate the transcription activity of NF-kappaB in the relative signal pathway (Ziesche et al., 2013). On the other hand, translocation of HDAC3 to the nucleus could promote NF-kappaB activity (Zhu et al., 2010); and HDAC3 may act as an intra-nuclear molecular switch of the NF-kappaB transcriptional response (Chen and Greene, 2004). However, whether translocation of HDAC3 occurs in chondrocytes and contributes to OA via the HDAC3/NF-kappaB pathway is still unclear. ROS, activate the NF-kappaB pathway, promoting inflammation and ECM degradation in OA (Lepetsos and Papavassiliou, 2016). HDAC3 also promotes ROS production in the inflammation response (Zhao et al., 2019). However, whether HDAC3 can regulate ROS production in OA has not been established.

This study aimed (1) to investigate the possible mechanism of physical therapy, which may modulate HDAC3 to prevent OA progression *in vivo*; (2) to identify the underlying mechanism of HDAC3 in OA *in vitro* and (3) to investigate the therapeutic effect of the HDAC3 inhibitor RGFP966 *in vivo* and *in vitro* (Malvaez et al., 2013; Schmitt et al., 2018). We identified a role for HDAC3 in the pathogenesis of OA, and we found that exercise therapy could attenuate inflammation in OA by modulating the HDAC3/NF-kappaB pathway. Our investigation also showed that RGFP966 exerts an anti-inflammatory effect in OA treatment.

## Materials and Methods

### Animal Model Construction

This study was carried out in accordance with the principles of the Basel Declaration and recommendations of U.K. Animals (Scientific Procedures) Act, Ethics Committee of China Medical University. The protocol was approved by the Ethics Committee of China Medical University. During experiments, we adhered to the 3R rules to ensure that rats were sacrificed comfortably. Adult male Sprague–Dawley (SD) rats (220–240 g; specific-pathogen-free) were purchased from HFK Bioscience, Co., Ltd. (Beijing, China). With a controlled temperature of 22 ± 2°C and 70% humidity, rats were housed with 12 h of light/dark cycles and fed with adequate food and drinking water. In order for the rats to adapt to the environment, they were fed for 1 week without any intervention. We anesthetized rats with 2% pentobarbital sodium by intraperitoneal injection (40 mg/kg) (Yang et al., 2018c), and shaved the hair around the bilateral knee joints with an electrical razor. Then, 50 μl saline containing 0.5 mg MIA (0.5 mg/50 μl) (Aike Reagent, Co., China) was injected into the intra-articular cavity per knee to construct the OA model according to previous literature (Udo et al., 2016), and 50 μl saline was injected per knee for the control group. The OA model was induced by intra-articular injection using a 50 μl micro-syringe through the lateral infrapatellar area into the distal femoral condylar space in an excessive knee flexion position.

### Exercise Protocol

We used rat-specific treadmill (ZH-PT, Zhongshidichuang Science & Technology Development, Co., Ltd., Beijing, China) for the experiments. Several generators were applied to the grid at the end of every lane, which could provide acoustic stimulation (80 dB), light stimulation (300 lx) and electric stimulation (100 Hz, 0.18 mA) to encourage rats when they failed to run on the treadmill. Firstly, rats were forced to run at a speed of 10 m/min for 10 min/day for 5 days to adapt to treadmill exercise. All rats (*n* = 50) were randomly divided into four groups: control group (CG); OA model group (OAG); MIA + low-intensity exercise group (OAL); MIA + moderate-intensity exercise group (OAM); and MIA + high-intensity exercise group (OAH).

### Formal Treadmill Exercise

**FIGURE 1:**
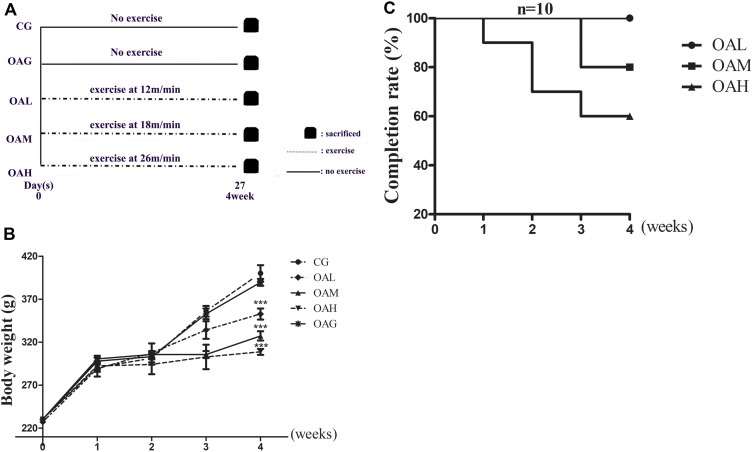
Protocols of different intensities of treadmill exercise on OA rats and completion rate, weight change of OA rats. **(A)** Different intensities of treadmill exercise on OA rats; speeds of 12, 18, and 26 m/min represent OAL, OAM and OAH respectively, and all groups were sacrificed after 4 weeks. **(B)** Weight change of rats in different groups. There was significant difference between exercise group and CG, OAG, whereas difference among OAL, OAM, and OAH was not significant; ^∗∗∗^*p* < 0.001; *n* = 6–10 rats for each group, means with SEM. **(C)** Completion rate of rats of different intensities of treadmill exercise. The completion was decreased along with increased intensity of treadmill exercise.

We set the speed for the OAH group at 26 m/min, 60 min/day, 5 days/week for 4 weeks; the OAM group at 18 m/min, 60 min/day, 5 days/week for 4 weeks and the OAL group at 12 m/min, 60 min/day, 5 days/week for 4 weeks. The treadmill incline was set as 5% (Sekiya et al., 2009) (Figure 1A). The OAL, OAM, and OAH groups began to run after MIA-injection for 24 h. The CG and OAG were kept in cages without any intervention. Body weight was recorded at regular intervals (Figure 1B). During the experimental period, rats that failed to run despite application of combined stimulation were defined as drop-outs and excluded from the experimental groups. Meanwhile, observation was required to ensure smooth execution of experiments, and the rats’ completion rate was calculated every week (Saito et al., 2017) (Figure 1C). Rats were sacrificed at the end of the experimental period.

### RGFP966-Injection in the OA Model

All SD rats (*n* = 40) were divided into four groups: CG, OAG, OA + RGFP966 group and RGFP966 group. The concentration of RGFP966 (MCE, HY13909) was 50 μM per knee. RGFP966 was first dissolved with DMSO and then diluted with saline (concentration of DMSO < 2%). 50 μM RGFP966 was administered by intra-articular injection per knee, 3 times/week for 4 weeks (Jia et al., 2016). CG was injected with same volume of saline; OAG was injected with 50 μl saline containing 0.5 mg MIA (0.5 mg/50 μl); OA + RGFP966 group was injected with RGFP966 after MIA injection for 24 h; RGFP966 group was only injected with RGFP966. The method of OA model construction and articular injection was the same as above mentioned. The rats were sacrificed after 4 weeks.

### Cell Culture

Primary rat chondrocytes were isolated from the knee joints of 4 week-old adult male Sprague–Dawley rats (100–150 g; specific pathogen-free). Primary rat chondrocytes were obtained by a two-steps enzymatic digestion. The cartilage was cut into 1 mm^2^ pieces followed by washing with PBS. Cartilage pieces were then digested with pronase K (4 mg/ml, Roche, Basel, Switzerland) for 1 h at 37°C and collagenase D (1.6 mg/ml, Roche) for 1 h at 37°C. Gentle mechanical vibration was applied in the whole digestion. Cell suspensions were centrifuged at 800 rpm for 5 min, and resuspended in PBS. The suspensions were filtered through a 100 μm mesh to exclude micro-tissue pieces. Undigested cartilage pieces were then treated with collagenase D according to the protocol mentioned above until remained cartilage pieces disappeared. Primary rat chondrocytes were then transferred into 25 cm^2^ culture flasks at a density of 5 × 10^6^ cells, and cultured with DMEM with low D-glucose (Hyclone, South Logan, UT, United States) supplemented with 10% FBS (Bioind, China) and 1% penicillin and streptomycin (Hyclone) at 37°C in a 5% CO_2_ incubator. After attachment of chondrocytes, culture medium was replaced. Chondrocytes were split in a 1:3 ratio when cell confluence reached 70%. Immunocytochemical staining of collagen II was used to identify the purity of chondrocytes. To guarantee the chondrocyte phenotype and accuracy of the subsequent experiments, primary rat chondrocytes at passage 3 were used. Cells were stimulated with IL-1β (R&D, Minneapolis, MN, United States) at a concentration of 20 ng/ml for 24 h, to obtain OA-like chondrocytes. To determine the role of RGFP966 in inflammation response in chondrocyte, chondrocytes were incubated with IL-1β (20 ng/ml) and RGFP966 (10 μM) simultaneously. Then, the chondrocytes were collected after 24 h incubation.

### X-Ray Imaging Observation

Knee joint images were captured by X-ray (MX-20, Faxitron X-Ray, Corp., Lincolnshire, IL, United States). Rats were anesthetized with pentobarbital sodium by intraperitoneal injection (40 mg/kg), and immobilized in the supine position with the bilateral ankles fixed on the tray by adhesive tape. The lens was focused on the rats’ knee with an appropriate focal length and the exposure time was set at 5 min to ensure clear images. The degree of OA was evaluated by imaging manifestation, including narrowing of joint space, and calcification changes of articular surface according to the X-ray image score system in previous literature (Wei et al., 2017). All images were measured by two investigators in a blinded manner.

### Histological Examination

Knee joints with intact articular capsules were harvested and fixed in 4% paraformaldehyde for 7 days at room temperature, followed by decalcification in 10% EDTA solution for 28 days at 37°C. EDTA solution refreshed every 3 days. Specimens were dehydrated in graded alcohol, made transparent in xylene and embedded in paraffin wax. Specimens were sectioned at 4.5 mm in the sagittal position with intact epiphyseal line in both the tibial and femur portions, and stained with hematoxylin and eosin (H&E) and Toluidine blue O. Histological sections were visualized using an Olympus BX53 microscope (Olympus, Tokyo, Japan). The degree of cartilage damage of the knee joints was evaluated according to OARSI scores and Mankin scores (Custers et al., 2007; Gerwin et al., 2010; Lee et al., 2011). The range of OARSI and Mankin scores was 0–24 and 0–14 points, respectively. Femoral and tibial cartilages were evaluated separately. OA was scored by two individuals in a blinded manner, using a validated histologic scoring system.

### Immunostaining

Sections were deparaffinized in xylene and graded alcohol and washed using cold PBS. Subsequent steps were performed in humidified conditions. Enzymatic antigen retrieval (AR0026, Boster, Biological Technology, Pleasanton, CA, United States) was used to repair antigens at 37°C for 30 min. After that, endogenous peroxidase activity was eliminated by 3% H_2_O_2_ for 30 min at room temperature followed by washing there times in PBS; 5% goat serum was used to block non-specific antigens. Next, sections were incubated with rabbit polyclonal anti-collagen II antibody (1:50, Abcam, ab34712), rabbit polyclonal anti-MMP-13 antibody (1:50, Abcam, ab39012), rabbit polyclonal anti-ADAMTS5 antibody (1:50, Bioss, bs3573R), rabbit polyclonal anti-NF-kappaB P65 antibody (1:50, Proteintech, 10745-1-AP) and rabbit polyclonal anti-HDAC3 antibody (1:50, Proteintech, 10255-1-AP) at 4°C overnight. After extensive washing there times with PBS, biotinylated secondary antibodies were added to the sections at room temperature for 30 min, followed by washing three times in PBS; sections were then incubated with streptavidin/horseradish peroxidase (S-A/HRP) (Zhongshan Goldenbridge Biotechnology, Co., China) at room temperature for 30 min according to the manufacturer’s protocol. Sections were visualized with diaminobenzidine (DAB) staining for 1 min and counterstained with hematoxylin for 5 min. Sections were dehydrated with graded alcohol and xylene, and sealed with neural gums. Images of sections were captured with optical microscopy (Eclipse Ci, Nikon, Japan) and optical density was measured with image analysis software Image-Pro Plus, version 6.0 (Media Cybernetics, Inc., Rockville, MD, United States). Rabbit IgG as the primary antibody was used as a negative control, and other steps were similar to the protocol mentioned above (Supplementary Figure S4). Three images of each slide were captured at a 400× magnification, and the average optical density represented relative expression of protein; NF-kappaB was calculated by percentage of positively-stained cell (Zhang et al., 2019).

### Western Blot Analysis

Cells and rat cartilage were lysed in RIPA buffer (25 mM Tris-HCl pH 7.6, 150 mM NaCl, 1% NP-40, 1% sodium deoxycholate, 0.1% SDS) with 1 mM PMSF (Beyotime, ST506). Lysates were centrifuged at 14000 *g*/min for 5 min at 4°C and supernatants were collected. Proteins in the cytoplasm and nucleus were isolated by using a Cytoplasmic and Nuclear Protein Extraction Kit (78833, Thermo Fisher, United States), according to the manufacturer’s instructions and previous study (Huang et al., 2014). Protein concentration was measured by the bicinchoninic acid assay (Beyotime, P0010). Equal amounts of protein (40 mg) were separated by sodium dodecyl sulfate polyacrylamide gel electrophoresis, and transferred to polyvinylidene difluoride membranes (Millipore, Bedford, MA, United States). Membranes were then blocked with 5% non-fat milk (w/v) for 2 h at room temperature followed by incubation with primary antibodies overnight at 4°C. The following antibodies were used: rabbit polyclonal anti-HDAC3 antibody (1:1000, CST, 3949S), rabbit polyclonal anti-collagen II antibody (1:10000, Abcam, ab34712), rabbit polyclonal anti-MMP-13 antibody (1:2000, Abcam, ab39012), mouse monoclonal anti-GAPDH antibody (1:4000, Proteintech, 60004-1-Ig), rabbit polyclonal anti- NF-kappaB p65 antibody (1:4000, Proteintech, 10745-1-AP), rabbit polyclonal anti-β-actin (1:1000, Proteintech, 20536-1-AP), rabbit polyclonal anti-H3 antibody (1:4000, Proteintech, 17168-1-AP). After washing three times with Tris-buffered saline with 0.1% Tween-20 (TBST), the membranes were incubated with horseradish peroxidase-conjugated secondary antibodies (Beyotime, China) for 2 h at room temperature. Membranes were then washed with TBST three times; bands were detected by enhanced chemiluminescence (Millipore), and quantified using Image J software (National Institutes of Health). β-Actin and H3 were used as the cytoplasmic and nuclear internal controls, respectively (Supplementary Figure S6).

### Co-immunoprecipitation (Co-IP) Assay

Chondrocytes were lysed in IP RIPA (Beyotime, P0013) with Protease inhibitor cocktail (Beyotime, P1005). Lysates were centrifuged at 14000 *g*/min for 5 min at 4°C and supernatants were collected. 500 μg protein lysates were incubated with 30 μl protein A/G PLUS-Agarose beads (SantaCruz sc-2003) and 8 μl anti-NF-kappaB p65 antibody (Proteintech, 10745-1-AP) at 4°C overnight. Next day, the beads were washed three times with lysis buffer and collected at 1500 rpm at 4°C for 5 min. The beads mixed with 30 μl 2× loading buffer for the SDS-PAGE electrophoresis.

### Quantitative Reverse Transcription Polymerase Chain Reaction (qRT-PCR)

Total RNA was isolated from primary rat chondrocytes and rat model cartilage tissue using RNAiso Plus (Takara, Shiga, Japan) and 1 μg RNA was reverse transcribed into cDNA using PrimeScript RT reagent Kit with gDNA Eraser (Takara) according to the manufacturer’s instructions. The polymerase chain reaction (PCR) reaction was completed with a SYBR Green PCR kit (Takara) using the Applied Biosystems 7500 Real-Time PCR System in triplicate per primer and per sample. Operation conditions were: 95°C for 30 s, 95°C for 5 s, 40 cycles of 60°C for 34 s. Relative mRNA expression was calculated by 2^–△△Ct^, which represented the fold-change with β-actin as the internal control. The following primers were designed by and purchased from Sangon, China (Table 1).

**TABLE 1:** Sequences of primers used for real-time PCR.

**Gene symbol**	**Sequence**
HDAC3 forward	5′-TTGAAGATGCTGAACCATGC-3′
HDAC3 reverse	3′-TGGCCTGCTGTAGTTCTCCT-5′
ADAMTS-5 forward	5′-TCCTCTTGGTGGCTGACTCT-3′
ADAMTS-5 reverse	3′-GGATGTGGTTCTCGATGCTT-5′
MMP-13 forward	5′-TGGTCCAGGAGATGAAGACC-3′
MMP-13 reverse	3′-GTGCAGACGCCAGAAGAATC-5′
Collagen II forward	5′-ACGCTCAAGTCGCTGAACAACC-3′
Collagen II reverse	3′-ATCCAGTAGTCTCCGCTCTTCCAC-5′
NF-kappaB p65 forward	5′-GGCTTCTATGAGGCTGAACTCTGC-3′
NF-kappaB p65 reverse	3′-CTTGCTCCAGGTCTCGCTTCTTC-5′
β-Actin forward	5′-CACCCGCGAGTACAACCTTC-3′
β-Actin reverse	3′-CCACACTACCACCCATACCC-5′

### Cell Counting Kit-8 (CCK-8) Test

Primary rat chondrocytes were seeded in two 96-well plates (5 × 10^3^ cells per well) and cultured for 1–2 days at 37°C with DMEM containing 10% FBS until cell confluency reached 70%. One plate was then used for culture with serum-free DMEM containing graded concentration of RGFP966 (0, 0.3, 0.6, 1.2, 2.5, 5, and 10 μM) for 24 h at 37°C. The other 96-well plate was used for culture with serum-free DMEM containing 10 μM RGFP966 cultured at 37°C for varying times (0, 6, 12, 24, 36, and 48 h). After RGFP966 treatment, 10 μl CCK-8 (Beyotime, C0042) dissolved in 90 μl serum-free DMEM was added to each well followed by incubation for 2 h at 37°C. The absorbance value of each well was measured at wave length of 450 nm using a spectrophotometer (Synergy H1, BioTek, United States). The entire experiment was repeated five times.

### Cellular ROS Production

Chondrocytes were seeded and cultured in six-well plates at a density of 1 × 10^5^ per well. When confluency of chondrocyte reached 80%, chondrocytes were stimulated with IL-1β (10 ng/ml) and/or RGFP966 (10 μM) with serum-free DMEM for 24 h. ROS production was measured by flow cytometry with dichloro-dihydrofluorescein diacetate (DCFH-DA) (S0033, Beyotime). Chondrocytes were harvested and incubated with 10 mM DCFH-DA for 30 min at 37°C in the dark, and then washed three times in PBS. The negative control group included serum-free DMEM with no DCFH-DA (Supplementary Figure S5). Finally, 10000 events were analyzed with a FACSCalibur flow cytometer (BD Biosciences, San Diego, CA, United States) with 488 nm excitation and 525 nm emission wavelengths (Jeong et al., 2019). The mean value of ROS intensity in each group was used to analysis difference by One-way analysis of variance.

### Statistical Analysis

Results are represented as mean ± SEM using GraphPad Prism 5. The Shapiro–Wilk and Levene tests were used to examine the normality and homogeneity of the results, respectively. One-way analysis of variance was used for the parametric analysis. Non-parametric data were analyzed by the Kruskal–Wallis *H*-test. *p*-Values of < 0.05 were considered statistically significant.

## Results

### Weight Change and Completion Rate of Rats in the Treadmill Exercise Program

As well-established, weight load can affect cartilage development; accordingly overload was also a cause of OA. We therefore detected weight changes of the rats in the program (Ernest and Kondrashov, 2018). Compared with the CG, the average weight of rats in the different exercise groups was significantly lower. Meanwhile, there were no differences in weight between the OAM and OAH groups, although OA symptoms were fewer in the OAM group compared to the OAH group (Figure 1B). We suggest that weight changes did not affect our experiments. According to the literature (Saito et al., 2017), we knew that not all rats could complete the exercise program due to intolerant intensity, especially in the OAH group. We found that all rats in the OAL group were able to complete the program; drop-out began at 3 week, and 80% of rats completed the program in the OAM group. Drop-out began at 1 week and 60% of rats completed the program in the OAH group (Figure 1C).

### Histological Changes of OA Affected by Treadmill Exercise

**FIGURE 2:**
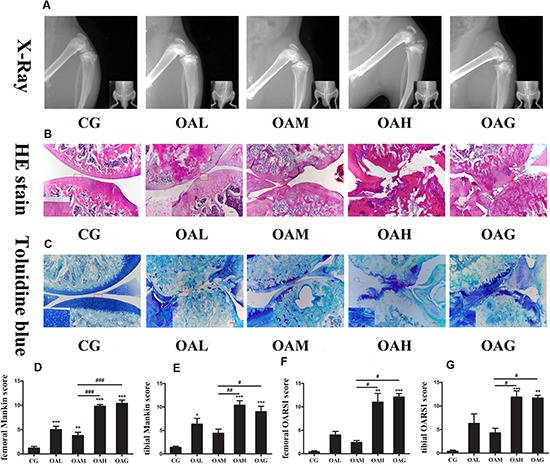
Histological changes of the articular joints of OA rats treated with different intensities of treadmill exercise. **(A)** X-ray image of articular joints of OA rats. Articular damage was severe in OAH and OAG, whereas the appearance was light in OAM. **(B)** H&E image of articular joints of OA rats. Articular surface was disappeared in OAH and OAG, whereas articular surface refreshed in OAM. **(C)** Toluidine blue O image of articular joints of OA rats. Cartilage erosion and staining reduction were in OAH and OAG, whereas compensatory regeneration of cartilage was in OAM. **(D)** Femoral Mankin score for articular joints. Femoral Mankin score of CG was lowest; OAM was lower than that of OAH and OAG. **(E)** Tibial Mankin score for articular joints. Tibial Mankin score of CG was lowest; OAM was lower than that of OAH and OAG. **(F)** Femoral OARSI score for articular joints. Femoral OARSI score of CG was lowest; OAM was lower than that of OAH and OAG. **(G)** Tibial OARSI score for articular joints. Femoral OARSI score of CG was lowest; OAM was lower than that of OAH and OAG; ^∗∗∗^*p* < 0.001; ^∗∗^*p* < 0.01; ^∗^*p* < 0.05 vs. CG, ^###^*p* < 0.001; ^##^*p* < 0.01; *^#^p* < 0.05 vs. OAM; *n* = 5 rats for each group, means with SEM.

We constructed the most suitable OA rat model successfully, according to our previous study (Yang et al., 2017), and then explored the effect of treadmill exercise on OA. We observed histological changes in the articular joints of OA rats in conditions of different exercise intensities. In macroscopic and imaging observations, we found cartilage damage and synovitis in the OAH group, similar to the OAG (Figure 2A), whereas the OAM was shown to alleviate OA symptoms. In X-ray examinations, imaging symptoms and score in the CG and OAM were lower than the OAG and OAH (Supplementary Figure S1). As for H&E and Toluidine blue O staining, we found cartilage surface erosion and chondrocyte death in the OAH group; nevertheless, OAM could refresh the cartilage surface and relieve chondrocyte death (Figures 2B,C). Meanwhile, the OARSI and Mankin scores of tibial and femoral cartilage were also lower in the OAM group than the OAH and OAG group (Figures 2D–G).

### The Expression of Relative Inflammatory Proteins/Genes in OA Treated With Treadmill Exercise

**FIGURE 3:**
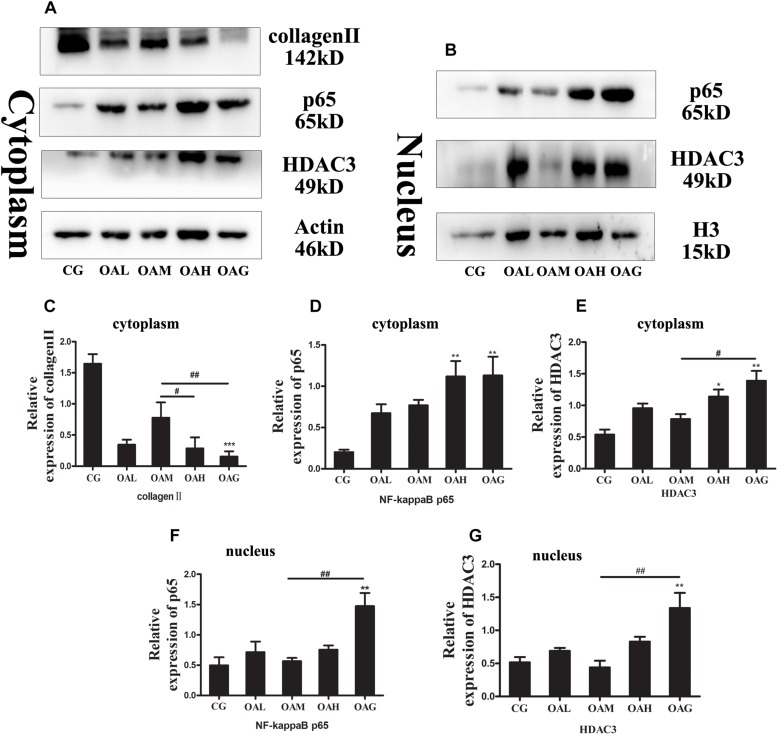
Osteoarthritis rats were treated with different intensities of treadmill exercise. Cytoplasmic and nuclear proteins were extracted from the articular cartilage of rats. Western blotting was performed to measure relative inflammatory proteins and HDAC3 in OA. The data was obtained in three separate experiments with β-actin as a cytoplasmic internal control, and H3 as a nuclear internal control. **(A,C)** Expression of collagen II in cytoplasm in CG was highest, and expression of collagen II in cytoplasm in OAM was higher than OAG and OAH. **(A,D)** Expression of NF-kappaB in cytoplasm in OAH and OAG was higher than CG, and the difference among OAM and OAH, OAG was not significant. **(A,E)** Expression of HDAC3 in cytoplasm in CG was lowest, and expression of HDAC3 in cytoplasm in OAM was lower than OAH and OAG. **(B,F)** Expression of NF-kappaB in nucleus in OAG was higher than CG and OAM. **(B,G)** Expression of HDAC3 in the nucleus in OAG was higher than CG and OAM; ^∗∗∗^*p* < 0.001; ^∗∗^*p* < 0.01; ^∗^*p* < 0.05 vs. CG, ^##^*p* < 0.01; ^#^*p* < 0.05 vs. OAM; *n* = 3 rats for each group, means with SEM.

**FIGURE 4:**
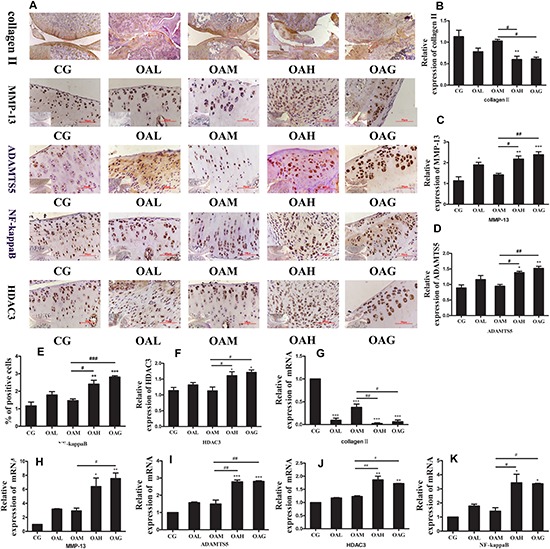
Osteoarthritis rats were treated with different intensities of treadmill exercise. Real-time PCR and immunohistochemistry (IHC) were performed to measure relative inflammatory genes/proteins in OA. **(A,B)** Expression of collagen II in CG and OAM were higher than OAH and OAG by IHC. **(A,C)** Expression of MMP-13 in CG and OAM were lower than OAH and OAG by IHC. **(A,D)** Expression of ADAMTS5 in CG and OAM were lower than OAH and OAG by IHC. **(A,E)** The percentage of NF-kappaB positive cell in CG and OAM were lower than OAH and OAG by IHC. **(A,F)** Expression of HDAC3 in CG and OAM were lower than OAH and OAG by IHC. **(A,G)** mRNA of collagen II in CG and OAM were higher than OAH and OAG by real-time PCR analysis. **(A,H)** mRNA of MMP-13 in CG and OAM were lower than OAG and mRNA of MMP-13 in CG was lower than OAH by real-time PCR analysis. **(A,I)** mRNA of ADAMTS5 in CG and OAM were lower than OAH and OAG by real-time PCR analysis. **(A,J)** mRNA of HDAC3 in CG and OAM were lower than OAH and OAG by real-time PCR analysis. **(A,K)** mRNA of NF-kappaB in CG and OAM were lower than OAH and OAG by real-time PCR analysis; ^∗∗∗^*p* < 0.001;^∗∗^*p* < 0.01; ^∗^*p* < 0.05 vs. CG, ^###^*p* < 0.001; ^##^*p* < 0.01; ^#^*p* < 0.05 vs. OAM; *n* = 3 rats for each group in IHC and *n* = 6 rats for each group in real-time PCR, means with SEM.

We identified the expression of OA-relative proteins treated with different intensities of treadmill exercise by immunohistochemistry (IHC) and western blotting. The expression of collagen II protein was increased in the OAM group compared to the OAH group or OAG (Figures 3A,C, 4A,B), whereas MMP-13 and ADAMTS-5 were decreased in the OAM group compared to the OAH group or OAG (Figures 4A,C,D). We also detected NF-kappaB expression in the cytoplasm and nucleus; nuclear expression of NF-kappaB was significantly lower in the OAM group than the OAH group and OAG according to IHC, whereas nuclear expression of NF-kappaB was lower in the OAM group compared to the OAG according to Western blotting; expression of NF-kappaB in the cytoplasm was also significantly decreased in the OAM group compared to the OAH group or OAG (Figures 3A,B,D,F, 4A,E). We found that expression of HDAC3 protein (both in the nucleus and cytoplasm) was significantly decreased in the OAM group compared to OAG (Figures 3A,B,E,G), and that the total HDAC3 protein was decreased in the OAM compared to OAH group and OAG (Figures 4A,F). We also detected mRNA changes by real-time PCR; collagen II mRNA level increased in the OAM group compared to the OAH group or OAG (Figure 4G); MMP-13 mRNA level were lower in the OAM group than the OAG (Figure 4H); ADAMTS5, HDAC3 and NF-kappaB mRNA levels were greater in the OAM group compared to the OAH group and OAG (Figures 4I–K).

### Histological Changes and Expression of Relative Proteins of OA Affected by RGFP966

**FIGURE 5:**
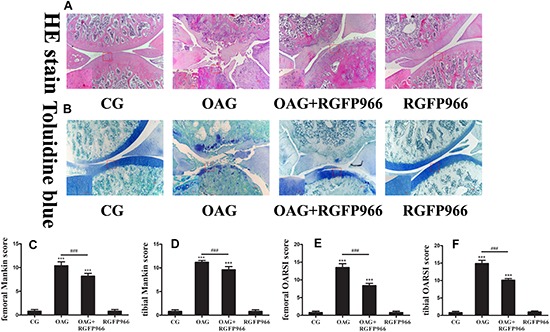
Histological changes of the articular joints of OA rats treated with RGFP966. **(A)** H&E image of articular joints of OA rats. Articular surface was damaged in OAG, whereas articular surface refreshed in OAG + RGFP966 group. **(B)** Toluidine blue O image of articular joints of OA rats. Cartilage damage and staining reduction were in OAG, whereas compensatory regeneration of cartilage was in OAG + RGFP966 group. **(C)** Femoral Mankin score for articular joints. Score of OAG + RGFP966 group was lower than that of OAG. **(D)** Tibial Mankin score for articular joints. Score of OAG + RGFP966 group was lower than that of OAG. **(E)** Femoral OARSI score for articular joints. Score of OAG + RGFP966 group was lower than that of OAG. **(F)** Tibial OARSI score for articular joints. Score of OAG + RGFP966 group was lower than that of OAG; ^∗∗∗^*p* < 0.001 vs. CG, ^###^*p* < 0.001 vs. OAG + RGFP966 group; *n* = 5 rats for each group, means with SEM.

To confirm the role of HDAC3 in OA *in vivo*, we treated OA rats with HDAC3-specific inhibitor RGFP966 by intra-articular injection for 4 weeks. In H&E and Toluidine blue O staining, the level of cartilage surface damage and chondrocyte death in the OAG + RGFP966 group was lower than the OAG (Figures 5A,B), which was also represented by OARSI and Mankin scores of femoral and tibial cartilage (Figures 5C–F).

**FIGURE 6:**
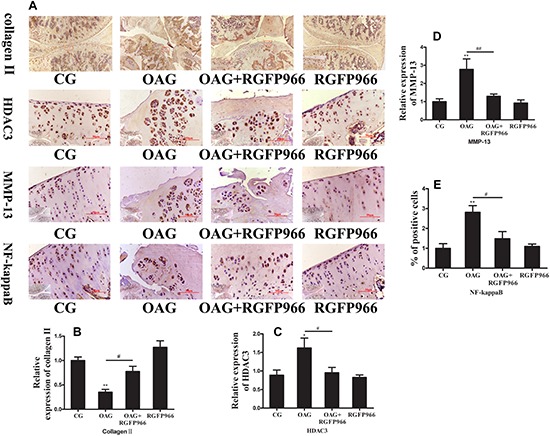
Osteoarthritis rats were treated with RGFP966. Immunohistochemistry (IHC) was performed to measure relative inflammatory proteins in OA. **(A,B)** Expression of collagen II in CG and OAG + RGFP966 group was higher than OAG by IHC. **(A,C)** Expression of HDAC3 in CG and OAG + RGFP966 group was lower than OAG by IHC. **(A,D)** Expression of MMP-13 in CG and OAG + RGFP966 group was lower than OAG by IHC. **(A,E)** The percentage of NF-kappaB positive cell in CG and OAG + RGFP966 group were lower than OAG by IHC; ^∗∗^*p* < 0.01; ^∗^*p* < 0.05 vs. CG, ^##^*p* < 0.01; ^#^*p* < 0.05 vs. OAG + RGFP966 group; *n* = 5 rats for each group in IHC, means with SEM.

We then detected the expression of OA-relative proteins in OA with intra-articular injection with RGFP966 by IHC. Collagen II in the OAG significantly was lower than the CG and OAG + RGFP966 group (Figures 6A,B), whereas MMP-13, percentage of NF-kappaB cells and HDAC3 in the OAG were significantly greater compared with the CG and OAG + RGFP966 group (Figures 6A,C–E). We therefore concluded that RGFP966 had a protective effect on OA *in vivo*.

### Isolation and Identification of Primary Rat Chondrocytes

**FIGURE 7:**
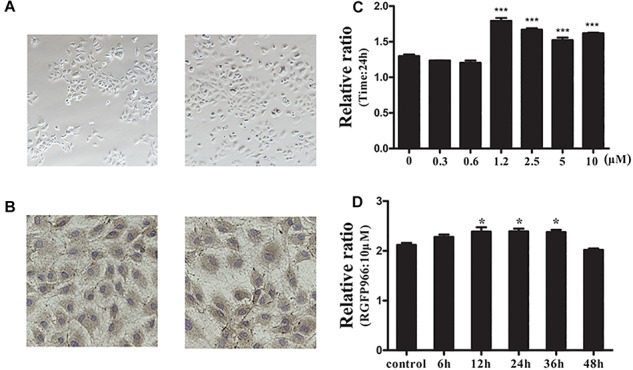
Isolation and identification of primary rat chondrocytes and the effect of the HDAC3 inhibitor RGFP966 on chondrocyte viability. **(A)** Primary rat chondrocytes were isolated from 4-week old male rats and the chondrocyte morphology was polygon. **(B)** Primary rat chondrocytes were stained with collagen II antibody for identification, almost all cells were stained with collagen II. **(C)** The effect of different concentrations of RGFP966 on chondrocyte viability with the same duration, RGFP966 with concentration range between 1.2 and 10 μM could promote chondrocyte viability for 24 h. **(D)** The effect of 10 μM RGFP966 on chondrocyte viability in a time-dependent manner, RGFP966 with time range between 12 and 36 h could promote chondrocyte viability with 10 μM concentration; ^∗∗∗^*p* < 0.001; ^∗∗^*p* < 0.01; ^∗^*p* < 0.05 vs. control group; *n* = 3, means with SEM.

To ensure accuracy of the subsequent experiments, we identified primary rat chondrocytes isolated from male rat knee cartilage. Under microscopic examination, the shape of normal chondrocyte was polygonal (Figure 7A). We also identified chondrocytes through IHC using the collagen II antibody (Figure 7B).

### The Effect of HDAC3 Inhibitor RGFP966 on Chondrocyte Viability

There has been little reported evidence about the effect of RGFP966 on chondrocyte. To identify the most suitable concentration of RGFP966, we firstly identified the changes in chondrocyte viability stimulated with different RGFP966 concentrations. We established a concentration range from 1 to 10 μM according to the literature (Leus et al., 2016; Correa et al., 2017). We found that RGFP966 can promote chondrocyte viability in a dose-dependent manner, and that 10 μM was the most suitable concentration (Figure 7C). We then investigated chondrocyte viability stimulated with 10 μM RGFP966 with different durations. We found that RGFP966 also promoted chondrocyte viability in a time-dependent manner and that 24 h was the most suitable time (Figure 7D).

### RGFP966 Attenuated Nuclear Location of NF-KappaB Mediated by HDAC3

**FIGURE 8:**
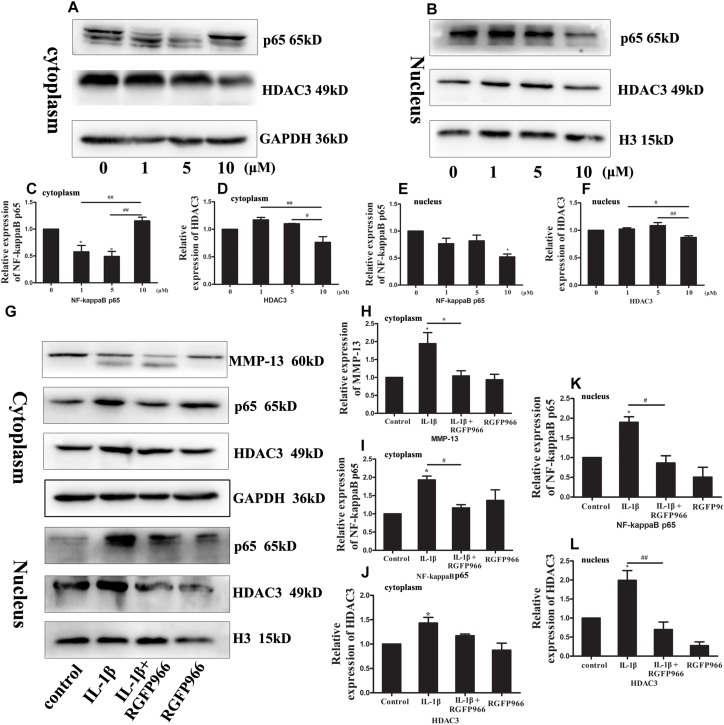
Western blotting was performed to measure relative inflammatory protein and HDAC3 expression in chondrocytes stimulated with RGFP966 and/or IL-1β. **(A,B)** Primary rat chondrocytes were stimulated with different concentrations of RGFP966 for 24 h, and cytoplasmic and nuclear proteins were extracted. NF-kappaB and HDAC3 levels were measured by western blotting. **(C,D)** Expression of NF-kappaB in cytoplasm in 1 and 5 μM group were higher than control and 10 μM group, and expression of HDAC3 in cytoplasm in 1 and 5 μM group were higher than 10 μM group **(E,F)** Expression of NF-kappaB in nucleus in 10 μM was lower than control group, and expression of HDAC3 in nucleus was lower than 1 and 5 μM group. ^∗^*p* < 0.05 vs. control group; ^##^*p* < 0.01; ^#^*p* < 0.05 vs. 10 μM group, *n* = 3, means with SEM. **(G)** Primary rat chondrocytes were stimulated with IL-1β (20 ng/ml) and/or RGFP966 (10 μM) for 24 h. Cytoplasmic and nuclear proteins were extracted and relative inflammatory proteins and HDAC3 were measured by western blotting. **(H–J)** Expression of MMP-13 and NF-kappaB in cytoplasm in IL-1β group was higher than control and IL-1β + RGFP966 group, and expression of HDAC3 in cytoplasm in IL-1β group was higher than control group. **(K,L)** Expression of NF-kappaB and HDAC3 in nucleus in IL-1β group was higher than control and IL-1β + RGFP966 group; ^∗^*p* < 0.05 vs. control group, ^##^*p* < 0.01; ^#^*p* < 0.05 vs. IL-1β + RGFP966 group; *n* = 3, means with SEM.

NF-kappaB is an important transcription factor that plays a crucial role in OA. More NF-kappaB in the nucleus means more activity and inflammation (Yang et al., 2018b). We investigated whether RGFP966 could reverse OA progression by regulating NF-kappaB activity. Firstly, we used different concentrations of RGFP966 to stimulate normal chondrocytes. We found that NF-kappaB expression in the cytoplasm of the 10 μM group was similar to the control group, and higher than the 1 and 5 μM groups, whereas NF-kappaB expression in the nucleus of the 10 μM group was lower compared to the other groups (Figures 8A–C,E). HDAC3 levels in the cytoplasm and nucleus of the 10 μM group also decreased significantly, compared with the other groups, while there was no significant difference between the 10 μM and control groups (Figures 8A,B,D,F). We then identified the effect of RGFP966 on IL-1β-stimulated chondrocytes. We demonstrated that MMP-13 expression increased in the IL-1β group, and decreased significantly after RGFP966 stimulation (Figures 8G,H). We found that IL-1β could elevate NF-kappaB expression in both the cytoplasm and nucleus of chondrocytes, whereas RGFP966 could reverse this effect (Figures 8G,I,K). We also found increased levels of HDAC3 in both the cytoplasm and nucleus upon IL-1β stimulation, and the increased HDAC3 expression in the nucleus could be reversed by RGFP966 (Figures 8G,J,L). We therefore conclude that RGFP966 could reduce HDAC3 expression in the nucleus in chondrocytes and inhibit the process of NF-kappaB relocation into the nucleus. Furthermore, we determined that HDAC3 could interact with NF-kappaB using co-IP, which suggested HDAC3 may regulate translocation of NF-kappaB by interaction with each other (Supplementary Figure S2).

### RGFP966 Could Inhibit ROS Production in IL-1β-Stimulated Chondrocytes

**FIGURE 9:**
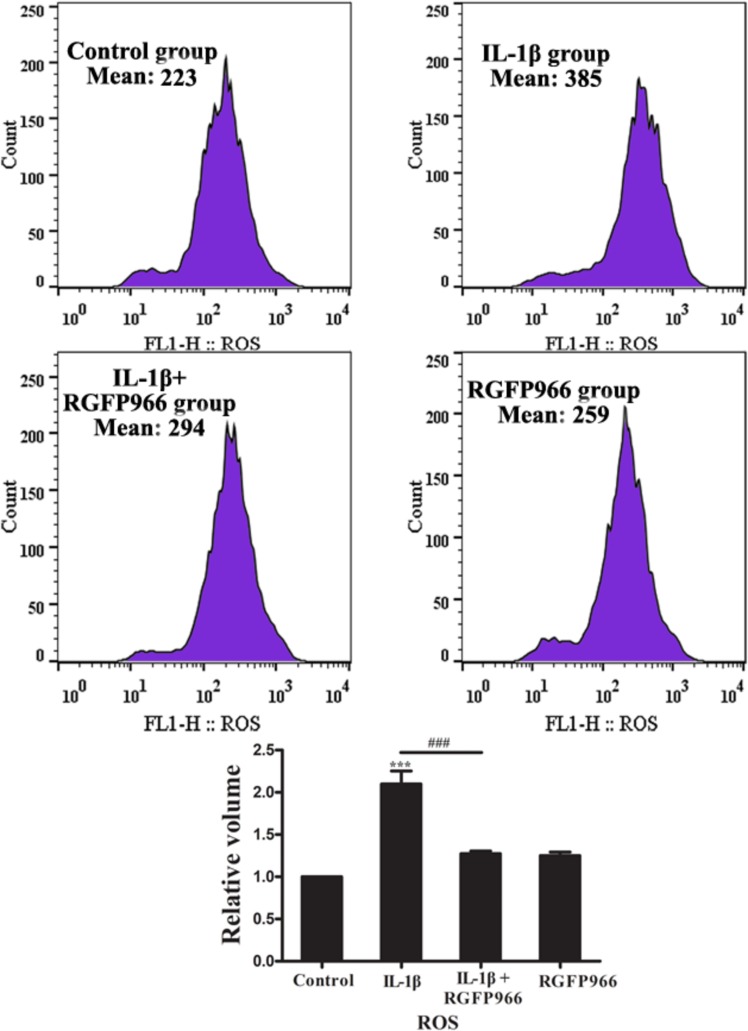
The effect of RGFP966 on ROS production in chondrocytes stimulated with IL-1β (20 ng/ml). Flow cytometry of ROS production in primary rat chondrocytes stimulated with IL-1β (20 ng/ml) and/or RGFP966 (10 μM) for 24 h, ROS production in IL-1β group was higher than control and IL-1β + RGFP966 group; ^∗∗∗^*p* < 0.001, ^∗^*p* < 0.05 vs. control group, ^###^*p* < 0.001 vs. IL-1β + RGFP966 group; *n* = 3, means with SEM.

Reactive oxygen species is a pro-inflammatory factor in many signaling pathways, and NF-kappaB is the downstream target of ROS (Yang et al., 2018a). It has been reported that ROS can also cause OA (Lepetsos and Papavassiliou, 2016). Upon IL-1β stimulation, the level of ROS in chondrocytes sharply increased, which was able to activate a series of inflammatory processes including the NF-kappaB signaling pathway. Conversely, RGFP966 could attenuate inflammation by inhibiting ROS production (Figure 9).

## Discussion

The etiology of OA is currently unclear, and consists of many factors such as genetics, environment, and habits (Martelpelletier et al., 2016). It is therefore important to determine the pathogenesis of OA. In our study, we found that HDAC3 is involved in the rat OA model in a time-dependent manner. In addition, moderate-intensity treadmill exercise could protect cartilage and inhibit the inflammatory response in OA. The protective effect of moderate-intensity treadmill exercise could be achieved by inhibiting HDAC3/NF-kappaB pathway. To further investigate the underlying mechanism of HDAC3 in OA, we successfully isolated primary rat chondrocytes followed by stimulation with IL-1β to mimic OA-like chondrocytes. We found RGFP966, an HDAC3-specific inhibitor, could alleviate IL-1β-induced inflammation and ROS by inhibition of nuclear translocation of NF-kappaB in chondrocyte. Furthermore, we also revealed RGFP966 could also alleviate inflammation in rat OA model.

Physical exercise has been considered as a new therapy for OA (McAlindon et al., 2014), our results indeed revealed that moderate intensity of treadmill exercise could prevent cartilage from damage, refresh OA cartilage and promote collagen II regeneration, consistent with previous studies (Na et al., 2014; Iijima et al., 2015). In addition, NF-kappaB is an important inflammatory transcription factor that promotes MMP-13 and ADAMTS5 expression resulting in cartilage matrix degradation and cartilage destruction (Roman-Blas and Jimenez, 2006; Wang et al., 2017; Yang et al., 2017). In our study, moderate-intensity treadmill exercise could reduce translocation of NF-kappaB into the nucleus in the rat OA model. It has been reported that elevated HDAC3 was found in the cartilage of OA patients (Meng et al., 2018). However, the role of HDAC3 in OA was still unclear. In the manuscript, we found that moderate-intensity treadmill exercise could decrease HDAC3 expression and promote nuclear export of HDAC3 in the rat OA model. Researchers have found that mechanical intervention or physical activity can regulate HDACs expression, also called mechanic-epigenetics. Appropriate exercise could inhibit HDACs expression (Sleiman et al., 2016), promote nuclear export of HDACs (Yuan et al., 2014) and inhibit HDACs activity (Spindler et al., 2014; Fu et al., 2019). However, the mechanism of reducing HDAC3 and the nuclear export of HDAC3 have not been demonstrated. According to previous articles, we suggested a possible mechanism, namely that degradation of HDACs may be caused by increased E3 ubiquitin ligase-induced degradation by the proteasome during exercise (McGee and Hargreaves, 2011). Exercise may also modulate phosphorylation of HDACs or an association between HDACs with other molecules, such as 14-3-3, which may also promote nuclear export of HDACs (Yuan et al., 2014). However, whether HDAC3 synthesis is also inhibited is unclear. Interestingly, high-intensity exercise was not able to protect cartilage in the rat OA model. The following mechanisms could be involved in this phenomenon: activation of osteoblasts may increase the subchondral bone mass to adapt to the increased physical load exposure, which could cause cartilage damage (Siebelt et al., 2014); the mechanobiology homeostasis of cartilage depends on the balance of hydrostatic stress and octahedral sheer stress. When overloaded on the knee, this balance is disturbed, resulting in mechanical damage of cartilage (Carter et al., 2004). Moreover, excessive ROS production may also contribute to OA with high-intensity exercise. Low levels of ROS are essential for normal cartilage and appropriate exercise could maintain the balance between oxidative and anti-oxidative system (Finaud et al., 2006). When cartilage is overloaded, the balance is broken and elevated ROS will cause injury to cartilage (Cooper et al., 2002; Baur et al., 2011). This phenomenon is similar to that *in vitro*, extreme axial compression increased oxidative stress in chondrocytes (Beecher et al., 2007). Meanwhile, the symptoms of OA were also alleviated by RGFP966 *in vivo*; the expression of MMP-13 and NF-kappaB decreased significantly in OA rats treated with RGFP966. Histological changes and the score system also represented the protective effect of RGFP966 *in vivo*.

**FIGURE 10:**
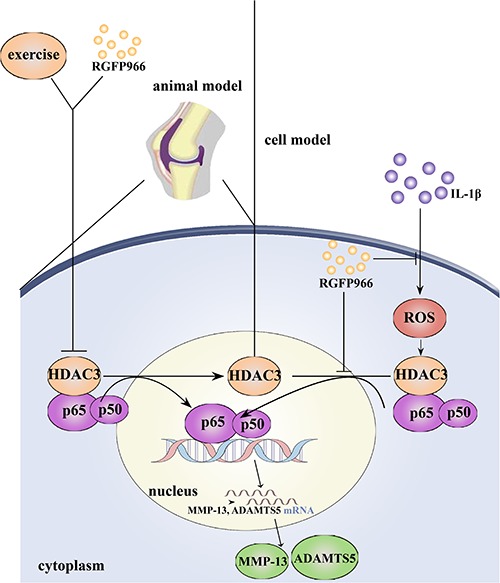
Schematic diagram of the mechanisms about the role of HDAC3/NF-kappaB pathway in pathogenesis and exercise therapy of OA. Moderate-intensity of exercise can inhibit nuclear translocation of HDAC3/NF-kappaB complex, leading to decreased expression of inflammatory proteins. Meanwhile, the HDAC3 inhibitor RGFP966 could also alleviate inflammation response in OA through HDAC3/NF-kappaB pathway *in vivo* and *in vitro*.

To further investigate the mechanism of HDAC3 in OA *in vitro*, we isolated primary rat chondrocytes and used RGFP966 at the basis of IL-1β stimulation to explore the effect of HDAC3 in OA. We found that RGFP966 could promote chondrocyte viability, and that high concentration of RGFP966 could significantly decrease the level of HDAC3 in the nucleus in normal chondrocytes, suggesting that the effect of HDAC3 on OA was dose-dependent. We also found elevated MMP-13 level and nuclear expression of NF-kappaB and HDAC3 was increased in chondrocytes stimulated with IL-1β, which could be reversed by RGFP966. When chondrocytes were stimulated with TNF-α instead of IL-1β we revealed the similar phenomenon (Supplementary Figures S7, S8). Accordingly, we suggest that HDAC3 is a pro-inflammatory factor in OA. The mechanism of HDAC3 in OA could be that HDAC3 regulates relative inflammatory gene expression (Angiolilli et al., 2017), and NF-kappaB activity (Leus et al., 2016). Interestingly, we found that the change in nuclear NF-kappaB and HDAC3 always occurred simultaneously. We therefore hypothesized that HDAC3 could associate with NF-kappaB translocation into the nucleus as a co-factor and promote NF-kappaB activity. To confirm this potential interaction, we investigated co-immunoprecipitation of HDAC3 with NF-kappaB. This finding was also supported by a database^1^ (Supplementary Figure S3). In previous studies, TNF-α stimulation was shown to enhance HDAC3-NF-kappaB complex translocation into the nucleus in 3T3-L1 adipocytes (Gao et al., 2006), and TNF-α also facilitated HDAC3 and NF-kappaB protein expression and nuclear translocation in A549 cells simultaneously in lung inflammation (Pooladanda et al., 2019). Furthermore, unphosphorylated NF-kappaB but not NF-kappaB phosphorylated at S536, was shown to bind to HDAC3 (Hu et al., 2004). A possible mechanism is that HDAC3 binds to unphosphorylated NF-kappaB in the cytoplasm and is translocated to the nucleus upon inflammatory stimulation (Pooladanda et al., 2019). Meanwhile, NF-kappaB separates from HDAC3 followed by S536 phosphorylation occurs. Another possible mechanism is that HDAC3 could change the acetylation level of NF-kappaB in the nucleus. CBP/p300 could promote NF-kappaB nuclear export through elevated levels of acetylation, whereas HDAC3 reverses this process (Kiernan et al., 2003). Meanwhile, RGFP966 might inhibit HDAC3/NF-kappaB pathway though above mentioned mechanism. However, the underlying mechanism of how HDAC3 can regulate NF-kappaB activity requires further investigation. ROS is a pro-inflammatory factor that can activate NF-kappaB in OA. We found that HDAC3 may also promote ROS production and activate NF-kappaB signaling via the ROS/NF-kappaB axis. It was reported HDAC3 promoted ROS production by regulating acetylation of Nrf2/ARE signaling (Chen X. et al., 2018). It is thus well-accepted that RGFP966 could inhibit ROS production in chondrocytes under IL-1β stimulation, which was demonstrated in a previous article (Zhao et al., 2019). We have therefore constructed a schematic diagram to summarize our experiments (Figure 10).

There are some limitations of our study. Firstly, it is not clear whether RGFP966 can substitute moderate exercise and there are possible side effects when administered to OA patients. Secondly, in this study, we only focused on changes in cartilage and chondrocyte. We should determine the mechanisms of cross-talk and feedback among other articular tissues (Loeser et al., 2012). Finally, it should be mentioned that we defined the different intensities of exercise according to the different percentages of VO_2max_ for SD rats (Bedford et al., 1979), but these results have not been translated into a clinical setting due to individual differences.

## Data Availability

The raw data supporting the conclusions of this manuscript will be made available by the authors, without undue reservation, to any qualified researcher.

## Ethics Statement

This study was carried out in accordance with the principles of recommendations of U.K. Animals (Scientific Procedures) Act, Ethics Committee of China Medical University. The protocol was approved by the Ethics Committee of China Medical University.

## Author Contributions

XZ conceived the study. YY developed the methodology. YW developed the software. LJ performed the formal analysis. YG investigated the results. JL provided the resources. HZ curated the data and drafted the manuscript. LB reviewed and edited the manuscript. All authors approved the final manuscript.

## Conflict of Interest Statement

The authors declare that the research was conducted in the absence of any commercial or financial relationships that could be construed as a potential conflict of interest.

## Footnotes

1 https://string-db.org

## Abbreviations

ADAMTS-5: a disintegrin and metalloproteinase with thrombospondin motifs-5
CG: control group
DMEM: Dulbecco’s modified Eagle medium
FBS: fetal bovine serum
GAG: glycosaminoglycan
HDACs: histone deacetylases
HDAC3: histone deacetylase 3
IHC: immunohistochemistry
IL-1 β: interleukin-1 β
MIA: monosodium iodoacetate
MMP-13: matrix metalloproteinase-13
NF-kappaB: nuclear factor-kappaB
OA: osteoarthritis
OAG: OA group
OAL: OA + low-intensity of treadmill exercise group
OAM: OA + moderate-intensity of treadmill exercise group
OAH: OA + high-intensity of treadmill exercise group
ROS: reactive oxygen species
TNF- α: tumor necrosis factor- α.
