# Supplementary material for: The Therapeutic Effects of Treadmill Exercise on Osteoarthritis in Rats by Inhibiting the HDAC3/NF-KappaB Pathway in vivo and in vitro
Source: Front Physiol. 2019 Aug 20;10:1060. doi: 10.3389/fphys.2019.01060 (PMC6710443; doi:10.3389/fphys.2019.01060)
Supplement: Supplementary file 1 [file Data_Sheet_1.docx]

**Supplement material**

**
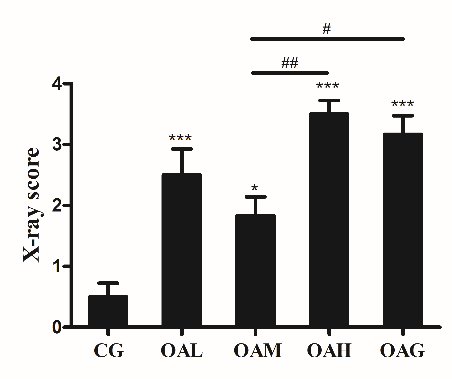
**

Figure S1. X-ray score of the articular joints of OA rats treated with different intensities of treadmill exercise. The X-ray scores in the OAH and OAG were higher than that in the OAM and CG, which represented that more cartilage damage occurred in OAH and OAG. ^***^*p*< 0.001; ^*^*p*<0.05 vs. CG, ^##^*p*<0.01; ^#^*p*<0.05 vs. OAM; n=5 rats for each group, means with SEM.


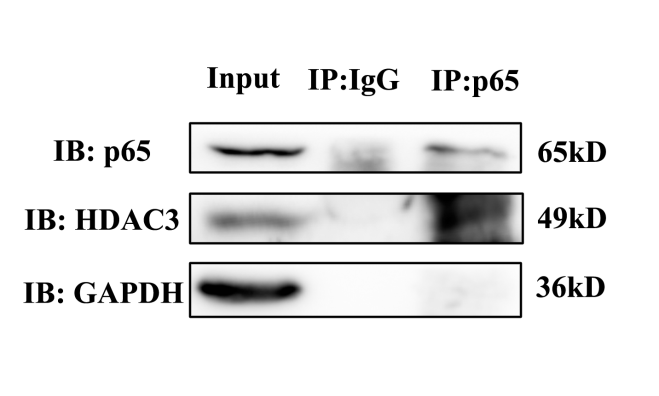


Figure S2. HDAC3 interacts with NF-kappaB p65 in chondrocytes. NF-kappaB p65 was immunoprecipitated from chondrocytes with an anti-NF-kappaB p65 antibody. The presence of NF-kappaB p65 and HDAC3 in these immunoprecipitates was evaluated with immunoblotting.


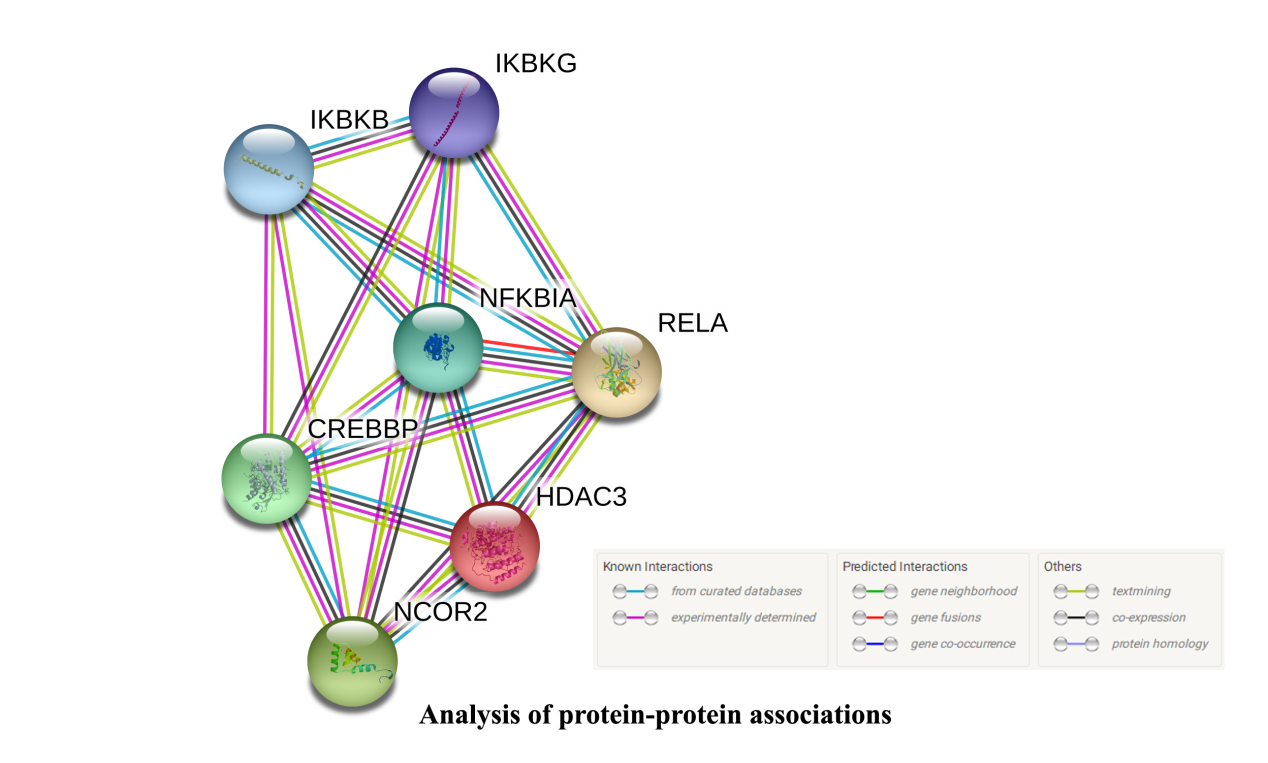


Figure S3. Analysis of protein-protein associations. It was reported that HDAC3 could interact with NF-kappaB (RELA) in the string database.


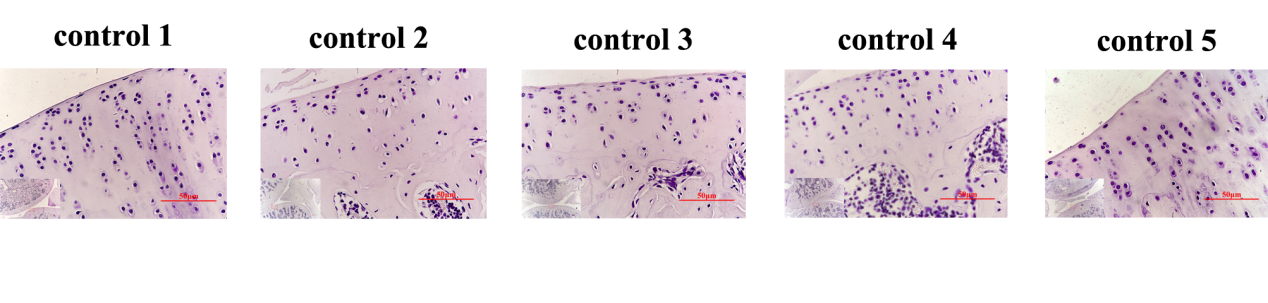


Figure S4. Isotype IgG controls for IHC staining. There was no positive staining in the isotype IgG control.

**
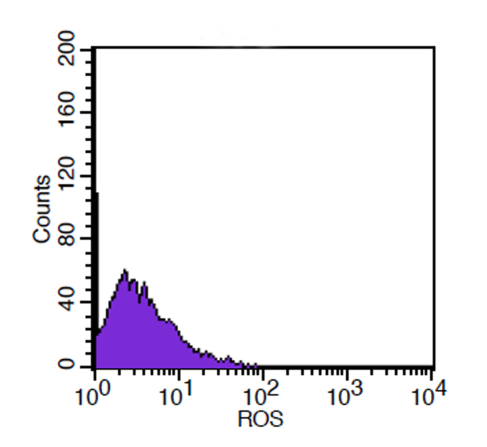
**

Figure S5. Isotype IgG control (negative control) for flow cytometric data of ROS. The negative control group was incubated with serum-free DMEM with no DCFH-DA. Meanwhile, the ROS in negative control was low.

**
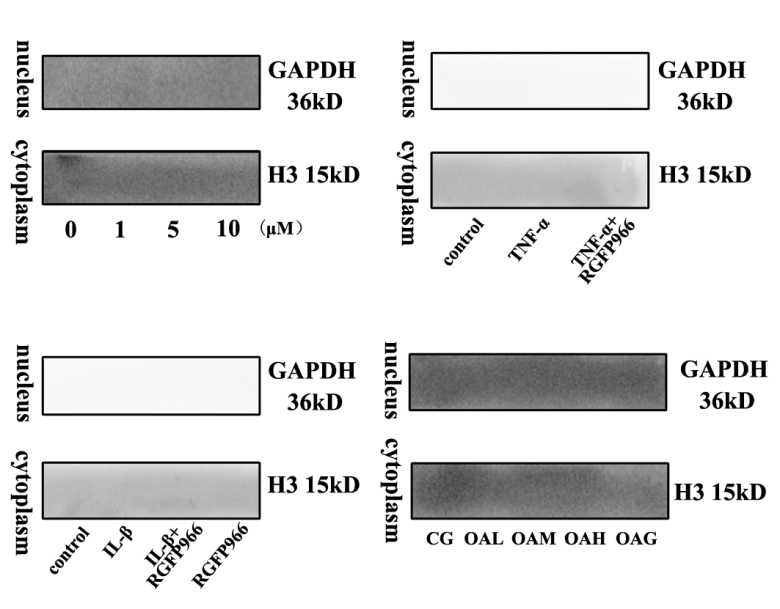
**

Figure S6. typical markers for cytoplasm or nucleus after fractionation. There was no cytosolic contamination in extraction of cytoplasmic and nuclear protein.

**the authors need to provide the negative control (cell without DCFH-DA), the difference of MIF in each group is not convincing, how to calculate the relative volume of ROS? The authors should provide fluorescence intensity**

Author action:


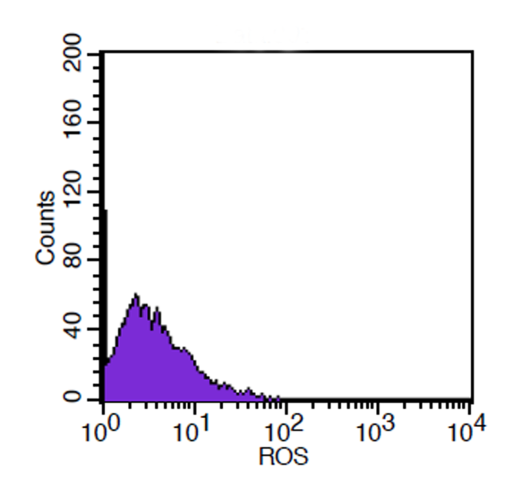


Mean fluorescence intensity of ROS for three replications, represented by mean ± SD:

Negative control group=7±2; Control group=196.7±29; IL-β group=400±16.5; IL-β+RGFP966 group =249±45.2; RGFP966 group=243±29.7.

**In the pathogenesis of OA, IL-1β and TNF-α are all involved in the ROS and NF-kappa B pathway, Whether RGFP966 also inhibited the TNF-α-mediated ROS production and NF-kappa B pathway?**

Author action:

Primary chondrocytes were incubated with TNF-α (20 ng/ml) (Beyotime, P6335) and RGFP966 (10 μM) simultaneously. Then, the chondrocytes were collected after 24 h incubation. The steps of protein extraction and Western Blot were the same with that mentioned in the manuscript.


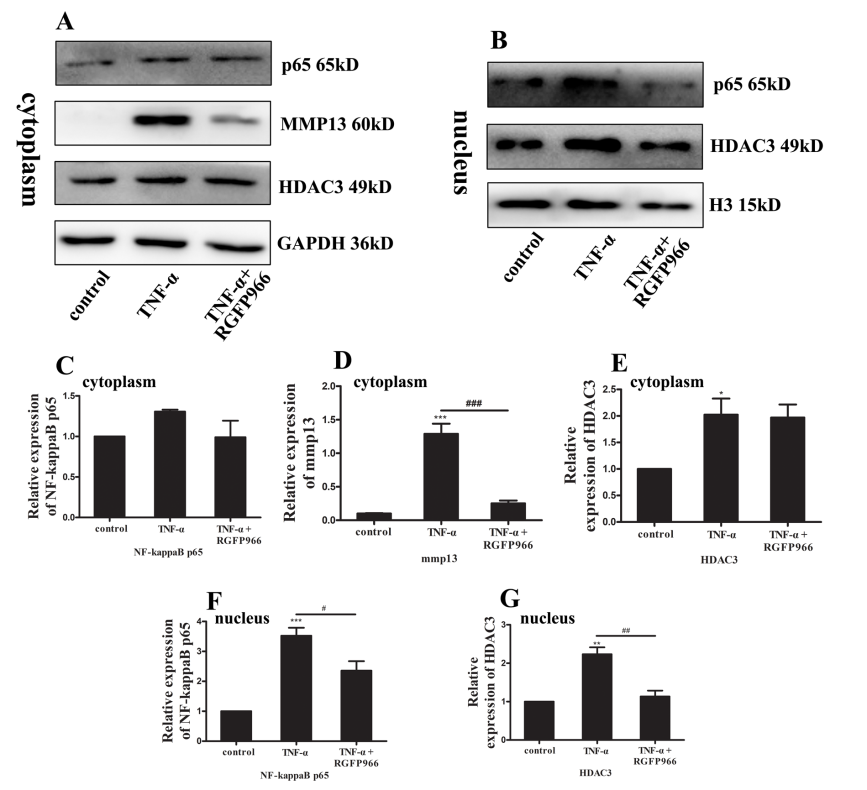


Figure S7. Western blotting was performed to measure relative inflammatory protein and HDAC3 expression in chondrocytes stimulated with RGFP966 and/or TNF-α. (A, B) Primary rat chondrocytes were stimulated with TNF-α (20 ng/ml) and/or RGFP966 (10 µM) for 24 h. Cytoplasmic and nuclear proteins were extracted and relative inflammatory proteins and HDAC3 were measured by western blotting. (C) Expression of NF-kappaB in cytoplasm in TNF-α group was higher than control and TNF-α+ RGFP966 group, whereas the difference was not significant. (D) Expression of mmp-13 in cytoplasm in TNF-α group was higher than control and TNF-α+ RGFP966 group. (E) Expression of HDAC3 in cytoplasm in TNF-α group was higher than control and the difference between control group and TNF-α+ RGFP966 group was not significant. (F, G) Expression of NF-kappaB and HDAC3 in the nucleus in TNF-α group was higher than control and TNF-α+ RGFP966 group; ^***^*p*<0.001; ^**^*p*<0.01; ^*^*p*<0.05 vs. control group; ^###^*p*<0.001; ^##^*p*<0.01; ^#^*p*<0.05 vs. TNF-α+ RGFP966 group; n=3, means with SEM.

Chondrocytes were seeded and cultured in six-well plates at a density of 1×10^5^ per well. When confluency of chondrocyte reached 80%, chondrocytes were stimulated with TNF-α (20 ng/ml) and/or RGFP966 (10 μM) with serum-free DMEM for 24 h. The following steps were the same with that mentioned in the manuscript.


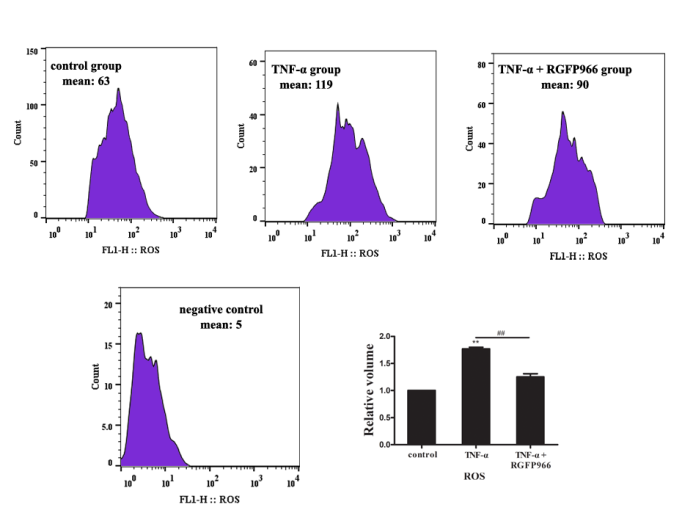


Figure S8. The effect of RGFP966 on ROS production in chondrocytes stimulated with TNF-α (20 ng/ml). Flow cytometry of ROS production in primary rat chondrocytes stimulated with TNF-α (20 ng/ml) and/or RGFP966 (10 µM) for 24 h, ROS production in TNF-α group was higher than control and TNF-α + RGFP966 group; ^**^p<0.01 vs. control group, ^##^*p*<0.01 vs. IL-1β + RGFP966 group.
